# Advances in Understanding the Role of Aloe Emodin and Targeted Drug Delivery Systems in Cancer

**DOI:** 10.1155/2022/7928200

**Published:** 2022-01-18

**Authors:** Gökçe Şeker Karatoprak, Esra Küpeli Akkol, Çiğdem Yücel, Özlem Bahadır Acıkara, Eduardo Sobarzo-Sánchez

**Affiliations:** ^1^Department of Pharmacognosy, Faculty of Pharmacy, Erciyes University, 38039 Kayseri, Turkey; ^2^Department of Pharmacognosy, Faculty of Pharmacy, Gazi University, Etiler, 06330 Ankara, Turkey; ^3^Department of Pharmaceutical Technology, Faculty of Pharmacy, Erciyes University, 38039 Kayseri, Turkey; ^4^Department of Pharmacognosy, Faculty of Pharmacy, Ankara University, Tandoğan, 06100 Ankara, Turkey; ^5^Instituto de Investigación y Postgrado, Facultad de Ciencias de la Salud, Universidad Central de Chile, 8330507 Santiago, Chile; ^6^Department of Organic Chemistry, Faculty of Pharmacy, University of Santiago de Compostela, 15782 Santiago de Compostela, Spain

## Abstract

Cancer is one of the important causes of death worldwide. Despite remarkable improvements in cancer research in the past few decades, several cancer patients still cannot be cured owing to the development of drug resistance. Natural sources might have prominence as potential drug candidates. Among the several chemical classes of natural products, anthraquinones are characterized by their large structural variety, noticeable biological activity, and low toxicity. Aloe emodin, an anthraquinone derivative, is a natural compound found in the roots and rhizomes of many plants. This compound has proven its antineoplastic, anti-inflammatory, antiangiogenic, and antiproliferative potential as well as ability to prevent cancer metastasis and potential in reversing multidrug resistance of cancer cells. The anticancer property of aloe emodin, a broad-spectrum inhibitory agent of cancer cells, has been detailed in many biological pathways. In cancer cells, these molecular mechanisms consist of inhibition of cell growth and proliferation, cell cycle arrest deterioration, initiation of apoptosis, antimetastasis, and antiangiogenic effect. In accordance with the strategy of developing potential drug candidates from natural products, aloe emodin's low bioavailability has been tried to be overcome by structural modifications and nanocarrier systems. Consequently, this review summarizes the antiproliferative and anticarcinogenic properties of aloe emodin, as well as the enhanced activity of its derivatives and the advantages of drug delivery systems on bioavailability.

## 1. Introduction

Cancer is an abnormality that occurs in the structure of the cell as a result of genetic or epigenetic changes. This negative directional change occurs uncontrollably and rapidly in cancer cells [1, 2]. In addition, cancer cells can escape from physiological suppressors and have metastasis properties with the mutations they undergo [2, 3]. Due to the rapid and active growth processes of tumor cells, cancer ranks second among the diseases that cause death worldwide [4]. Despite significant advances in cancer research, the development of multidrug resistance seen in many cancer patients in the last few decades reduces the success of chemotherapy [5, 6]. However, conventional cancer treatment consists of chemotherapy, radiation, and surgery and is often associated with morbidity, and cure rates are inadequate in several types of cancer [7–9].

Natural resources, which have been seen as potential drug candidates in recent years, have attracted great interest in both reducing symptoms and prolonging posttreatment survival in cancer patients [10]. Anthraquinones, which occupy a wide range of secondary metabolites in plants, are compounds with great structural diversity, pronounced biological activity, and low toxicity. They are mostly present in Fabaceae, Liliaceae, Polygonaceae, Rubiaceae, Rhamnaceae, and Scrophulariaceae families [11]. Aloe emodin (1,8-dihydroxy-3-hydroxymethyl-anthraquinone), which belongs to the anthraquinones as one of the class from quinone family of the polyketide group characterized by an amazing structural diversity of compounds, is a naturally occurring phenolic compound mainly found in *Aloe*, *Rheum*, and *Rhamnus* genera plants. This compound displays a variety of biological activities that include laxative, anti-inflammatory, immunomodulatory, wound healing, and antineoplastic activities [12].

Anthraquinones inhibited growth of tumor cell lines derived from the lung [13], breast [14, 15], colon [16], leukemia [17, 18], prostate [19], and cervix [20]. Moreover, the structural similarity of anthraquinone aglycons to anthracyclines as well as recognized anticancer drugs permits to speculate on their probable activities against cancer. Today, anthraquinone group drugs, which are used extensively in various cancer types, interact with DNA strands and target topoisomerase because of their planar tricyclic structure. However, it is also stated that cells develop a resistance mechanism against these natural anthraquinone compounds and remove them from the environment by different excretion routes. For this purpose, the idea that adding different side chains to the anthraquinone skeleton will increase its therapeutic effects and that new anthraquinone derivatives can prevent the development of resistance in cancer cells has gained importance [5, 6].

This review reports the presented data on the mechanisms of action of aloe emodin and summarizes main studies describing their anticancer activity, either alone or as main constituents of plant extracts, from the recent years.

## 2. Methodology

A search for peer-reviewed articles published before June 2021 was performed in Web of Science, PubMed, MEDLINE, and Google to identify studies observing the effects of aloe emodin. Search terms used were (“aloe emodin” or “anthraquinone” or “natural compound” or “natural product”) and (“cancer” or “apoptosis”). Plant names, authorities, and families were confirmed using http://www.tropicos.org/sites and http://www.theplantlist.org/. All titles/abstracts of the occasioning articles were appraised manually. For related abstracts, full articles were achieved and reviewed.

## 3. Chemical Properties of Aloe Emodin

Anthraquinones, as secondary metabolites, have an anthracene structure consisting of three benzene rings with a ketone group in positions 9 and 10 as core skeleton and substitution with different variety of functional groups such as -OH, -CH3, -OCH3, –CH2OH, –CHO, –COOH, and their derivatives. The anthraquinone molecules that contain hydroxyl groups are defined as hydroxyanthraquinones. 1,8-Dihydroxyanthraquinones substituted with the hydroxyl groups from the C-1 and C-8 carbons display acidic properties of which comparable to that of carboxylic acids since they have similar structures. They can be found as their free aglycone forms and as glycosides in the nature, in plants, lichens, insects, and higher filamentous fungi. These glycosides are produced by bonding one or more monosaccharide molecules, commonly glucose or rhamnose, to the aglycone through the hydroxyl group as O-glycoside linkage [21, 22].

**Figure 1:**
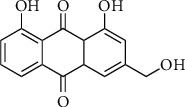
Chemical structure of aloe emodin.

Aloe emodin (1,8-dihydroxy-3-hydroxymethyl-anthraquinone), which belongs to the anthraquinones as one of the class from quinone family of the polyketide group characterized by an amazing structural diversity of compounds, is a naturally occurring phenolic compound mainly found in *Aloe*, *Rheum*, and *Rhamnus* genera plants. As shown in Figure 1, aloe emodin has a 9,10-anthraquinone skeleton and a hydroxymethyl group [11, 23].

**Figure 2:**
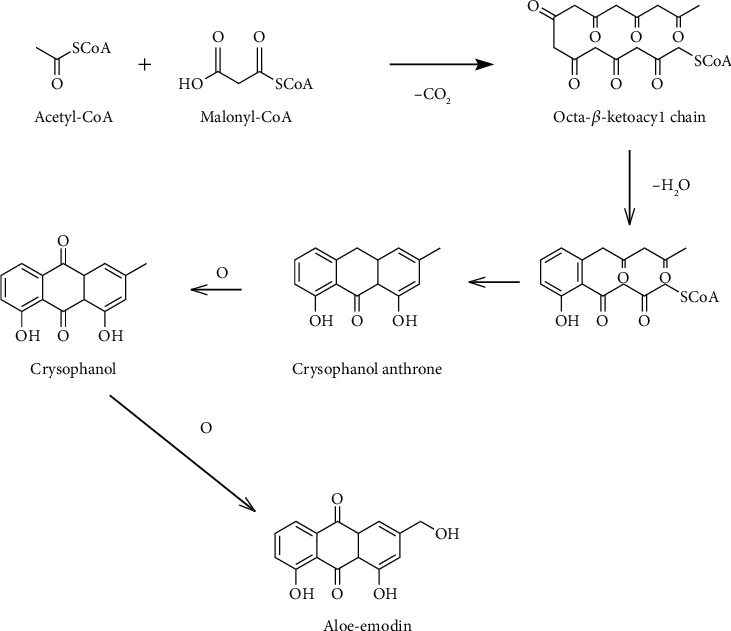
Biosynthetic pathway of aloe emodin.

Anthraquinone biosynthetic pathways have been described by two different main routes in plants as follows: (1) the polyketide pathway, which leads to production of polyketide anthraquinones with two rings hydroxylated by cyclization of the intermediate chain of octa-*β*-ketoacyl-CoA, by the addition of an acetyl-CoA to three malonyl-CoA units, and (2) the shikimate or chorismate/o-succinylbenzoic acid way, which occurs by the addition of succinoyl benzoic acid, obtained from shikimic acid and *α*-ketoglutaric acid, to mevalonic acid. This pathway resulted in the occurrence of only one hydroxylated ring containing anthraquinones. Anthraquinones have tricyclic aromatic ketones synthesized by the polyketide pathway. Several anthraquinones are produced as acetate-derived structures that aloe emodin is one of the excellent examples among them. Aloe emodin synthesized by polyketide pathway is exhibited in Figure 2 [21].

## 4. Anticancer Activity of Aloe Emodin

### 4.1. Effects on Cell Cycle Arrest of Aloe Emodin

Studies have displayed that the antiproliferative effect of aloe emodin on glioma, neuroblastoma, breast, gastric, oral mucosa, colorectal, cervical, prostate, liver, and leukemia cancer cells, as well as healthy keratinocyte cells, is concentrated and time-dependent. Detailed information on the results of the research has been given.

In the U87 glioma cell line, it was shown that aloe emodin had an antiproliferative effect. With the MTS test evaluating the cell proliferation, it was revealed that aloe emodin has a dose-dependent vitality-reducing effect between 0 and 80 *μ*g/mL. IC50 values for 24, 48, and 72 hours were 58.6, 25.0, and 24.4 *μ*g/mL, respectively [24]. In a different study during the treatment with aloe emodin, DNA content and distribution were examined in SJ-N-KP neuroblastoma cells, and the effect on proliferation was examined for 48 hours. According to the results, it has been stated that the induction of cell death may be related to the induction of DNA damage. The same study also found that aloe emodin did not affect the proliferation of normal fibroblasts and hemopoietic progenitor cells [25]. SVG (transformed glial cell) and U-373MG cells (human glioma) were treated with aloe emodin, and proliferation was examined with Trypan blue assay at 24, 48, 72, and 96 hours. The viability decreasing effect of aloe emodin for SVG and U-373MG was found as follows, respectively: 71–98% and 88–100% [26].

Different studies have been done to evaluate the antiproliferative effect of aloe emodin in breast cancer cell lines. In MDA-MB-453 and MCF-7, treatment of aloe emodin 6 to 100 *μ*M concentration significantly inhibited the MCF-7 proliferation in a dose-dependent manner but had a moderate effect on MDA-MB-453. The different effects in these two cell lines were attributed to Er*α* protein, which is initiated by estrogen and supports the initiation and progression of breast cancer [27]. Tseng et al. described that aloe emodin facilitates the cell death induced by tamoxifen and increases the antiproliferative activity of tamoxifen by hindering the PI3K/mTOR and Ras/ERK pathways in MCF7 cells [28]. The effects of aloe emodin on telomerase inhibition, which acts a crucial role in proliferation in different breast cancer cell lines (MDA-MB-453, MDA-MB-231, and MCF7), have also been studied. Telomerase inhibition in cancer cells causes telomere attrition, suppression of cell proliferation, and apoptosis. Results demonstrated that aloe emodin stabilized the G-quadruplex structure, interrupting the interaction between telomere and telomerase, shortening telomere length, and thus suppressing cancer cell proliferation [29]. Photosensitizers show specific toxicity by inducing intracellular radical oxygen species, so the photocytotoxicity of aloe emodin in MCF7 cells was evaluated for its photosensitizing effect. Inhibition of cell proliferation has been demonstrated after photodynamic application with different concentrations of aloe emodin and light doses [30].

The first of the studies on gastric cancer cell lines was performed in MGC-803 and SGC-7901 cell lines. Aloe emodin was applied to both cell lines at a concentration of 2.5, 5, 10, 20, and 40 *μ*M. It has been noted to exhibit stronger inhibition against the MGC-803 cell line and to have a long-lasting antiproliferation effect on SGC-7901 and MGC-803 [31]. In the second study, the BGC-823 cell line was used, and a dose-dependent antiproliferative effect was shown by aloe emodin [32]. In the study of Chihara et al., 0.05 mM aloe emodin was found to inhibit the proliferation of the MKN45 cells [33].

The lethal effects of aloe emodin at a concentration of 10, 20, 30, and 40 *μ*mol/L and laser with different radiation have been tested on KB cell line, which is an oral mucosa cell line. Aloe emodin treatment with photodynamic therapy caused blocking of cell proliferation in the G1 phase [34]. Various concentrations of aloe emodin in the range of 25-225 *μ*M inhibited proliferation in oral squamous cancer line (SCC15), and the half-maximal inhibitory concentration was found 60.90 *μ*M [35]. In the study of Chang et al., the esophageal cancer cells (TE1) were used to elucidate the cytotoxic effect mechanism of aloe emodin. In this study, aloe emodin was noted to inhibit TE1 cell proliferation in a concentration-dependent manner, at doses <20 *μ*M, lower than previously reported [36].

The antiproliferative effect of aloe emodin isolated from *Rheum ribes* was evaluated on human glioblastoma cell line (U373), human breast carcinoma cells (MCF-7), and human colorectal cancer cells (HT-29) between the concentration range of 1-100 *μ*g/mL. A dose-dependent antiproliferative effect was detected in all cell lines. The IC50 values of aloe emodin were 18.59, 16.56, and 5.38 *μ*g/mL in U373, MCF-7, and HT-29 cell lines, respectively, at 48 h treatment [37]. It has also been informed to reduce viable cell amount in a concentration-dependent manner in the range of 2.5-40 *μ*mol/L in the cervical cancer cell line (HeLa) [38].

In prostate cancer cell line (PC3), aloe emodin was studied at 2.5-15 *μ*M concentrations and has been found to inhibit proliferation and anchorage-independent growth of the androgen refractory prostate cancer cell line. It was also stated that it inhibited cell growth by reducing protein kinase B (Akt) phosphorylation, which is associated with tumorigenic potential [39].

Aloe emodin exerted different antiproliferative mechanisms in Hep 3B and Hep G2 liver cancer cell lines. Inhibition of cell proliferation was related to induced p53 (tumor protein p53) and p21 (inhibitor of cyclin-dependent kinase) expression in Hep G2 cells and the p21-dependent manner in Hep 3B cells [40]. Aloe emodin exerted an antiproliferative effect in Huh-7 hepatoma cell line with an IC50 value of approximately 75 *μ*M [41].

Promyelocytic HL-60, human myeloid leukemia U937, myelogenous leukemia K-562, and Burkitt's lymphoma P3HR-1 cells were used in studies evaluating the effects of aloe emodin on leukemia cell lines. In the HL-60 cell line, it was declared that the antiproliferative property of aloe emodin was related to the activation of caspase-3 [42]. In the U937 cell line, the reduction of cell proliferation was 70.45% after 72 h at 50 *μ*M concentration [43]. In recent times, Kuruca et al. presented the cytotoxicity of aloe emodin on HL-60, K-562, and P3HR-1 leukemia cell lines. The IC50 value of aloe emodin against K-562 was 60.98 *μ*M, 20.93 *μ*M for HL-60, and 28.06 *μ*M for P3HR-1 [44].

Similar to its effects on cancer cells, it was the first time that Popadic et al. illuminated the antiproliferative effect on normal keratinocyte cells. Although it was studied at low doses of 1.25, 2.5, and 5 *μ*M, a dose-dependent antiproliferative effect was observed [45]. Aloe emodin showed specific toxicity in the Merkel cell carcinoma (MCC) line defined as an aggressive, highly malignant, and undifferentiated carcinoma of the skin. A statistical significance was found for aloe emodin at concentrations of 10 *μ*mol and higher in the cell line (*p* < 0.05; *p* < 0.001) [46].

Cell proliferation and cell cycle progression are linked to the expression of genes that play a role in growth control. The cell cycle in which eukaryotic cells replicate themselves consists of the M (mitotic) phase, a G1 phase, an S phase, and a G2 phase. The G-M and G-S checkpoints and the metaphase-anaphase checkpoint are loop points that control the cell cycle. While the cell cycle is controlled by cyclin-dependent kinases and cyclins, cell homeostasis is maintained by proliferation, growth arrest, and apoptosis. When the cycle works properly, the cycle regulatory proteins control cell growth and induce the death of damaged cells, thus acting as the body's own tumor suppressor. Genetic mutations in the cell cycle can lead to uncontrolled cell proliferation, leading to carcinogenesis or tumor development [47, 48]. Numerous studies examining the cytotoxic properties of aloe emodin and its effects on the cell cycle were also investigated. The results of these studies are summarized in Table 1.

When researches are examined, it is understood that the mechanism of action of aloe emodin, which acts on cells, varies according to cell types. Gastric cancer (SGC-7901), promyelocytic leukemia (HL-60), glioma (U251), oral cancer (KB), human bladder cancer (T24), nasopharyngeal carcinoma (NPC), cervical cancer (HeLa), murine melanoma (B16), skin cancer (A431 and SCC25), and colon cancer (WiDr) cell lines were arrested at the G2/M phase by aloe emodin. The results show that the cyclin B1 and A levels expressed at high levels in the S to G2/M phases are significantly changed by aloe emodin. Esophageal cancer (TE1), malignant glioma (U87), tongue squamous cancer (SCC-4), pharyngeal squamous carcinoma (FaDu), human glioma (U-373MG), and transformed glia (SVG) cell lines were arrested at the S phase. Cyclin D1, cyclin B1, cyclin A, p53, p21, and p27 levels were affected by aloe emodin. Hep G2 hepatoma and MKN45 gastric cancer cell lines were arrested at G0/G1 phase, and an increment in p53 accumulation and CDK inhibitor expression was observed [59].

### 4.2. Effects on the Apoptosis Induction of Aloe Emodin

**Figure 3:**
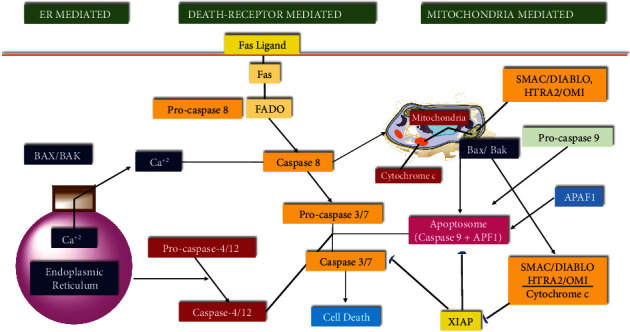
Pathways of apoptosis and regulation mechanism (adapted from reference [59]).

The progress of apoptosis is developmentally and homeostatically important in complex biological systems. Disorder or failure of normal apoptotic mechanisms contributes to the transformation of cells and ensures an advance to cancer cells for growth. DNA fragmentation, chromatin condensation, cell shrinkage, and the activation of caspases are characterized by apoptosis [60]. Aloe emodin's ability to induce apoptosis in different cancer cells is one of the main reasons for its anticancer effect. The mechanism by which this anthraquinone derivative induces apoptosis has been explained in different ways. However, it is generally stated that the mitochondrial apoptotic pathway is activated in cancer cell lines because of aloe emodin application. Apoptotic pathways and their regulation are illustrated in Figure 3.

Studies on various lung cancer cell lines have shed light on the apoptosis mechanism of aloe emodin. In the first of these studies, activation of caspase-3, defined by an increase in cytochrome c (Cyt c) and cleavage of its preform was observed in the CH27 cell line. It has also been claimed that aloe emodin induces changes of PKC isozymes in both H460 and CH27 cells and thus induces apoptosis [61]. In a different study, apoptosis of CH27 cells was related to the translocation of Bax and Bak from cytosolic to particulate fractions, which modulated the expression of Bcl-2 (BclX (L), Bak, and Bag-1) proteins [62]. The apoptosis of the H460 cell was found to be induced by nucleophosmin release from the nucleus to the cytosol and nucleophosmin degradation [63]. It was stated that apoptosis in the same cell line was caused by the 40 *μ*m dose of aloe emodin that caused aggregation of DNA [64]. Yeh et al. explained that aloe emodin-induced apoptosis of the same cell line was due to modulation of protein kinase, caspase-3, Bcl-2, PKC, and p38 protein expression due to extracellular adenosine 3′,5′-cyclic monophosphate (cAMP) [65]. Again, with the study performed in the H460 cell, the mechanism that causes apoptosis is that the activity of mitochondria is disrupted by this anthraquinone. In addition, it was observed that the protein expression of 70 kDa heat shock proteins (HSP70), 150 kDa oxygen-regulated protein, and protein disulfide isomerase, which are endoplasmic reticulum chaperones, and 60 kDa heat shock protein (HSP60), is increased by aloe emodin and caused apoptosis [66]. In a later study, photoactivated aloe emodin rapidly opened the mitochondrial permeability transition pore and induced H460 cell death by causing changes in apoptosis-associated proteins (*α*-actinin, p38, and feat shock protein 27) expression. Besides, a significant reduction in Bcl-2 protein levels and proform caspase-3 and caspase-7 protein expression levels and increase in proform of caspase-8 and caspase-9 were determined after photoactivated aloe emodin treatment [67].

Mechanism of apoptosis in human glioma (U-373MG) cell of aloe emodin was researched by Acevedo-Duncan et al. It was determined that PKC isozyme levels decreased after aloe emodin treatment. It also induced caspase-7 activation and caused an important reduction in the expression of survivin, which is necessary for cell viability [26]. An article investigating the effect of aloe emodin on mitochondrial membrane potential, published by Ismail et al. measuring the membrane potential of mitochondria, which has a regulatory role in initiating apoptosis, is also important for intrinsic caspase/proapoptotic activation [68]. According to the confocal microscopic examinations, U87 (human glioblastoma) cells treated with aloe emodin showed green fluorescence, demonstrating a collapse in mitochondrial membrane potential. With agarose gel electrophoresis, a typical apoptotic oligonucleosomal fragmentation was detected [24]. By transmission electron microscopy (TEM) analysis, it was shown that apoptosis (SJ-N-KP) in neuroblastoma cells was in the form of cell shrinkage, membrane bubbles, and nuclear fragmentation [25]. Pecere et al., in a later study, used neuroblastoma cell lines (SK-N-BE(2c) and SJ-N-KP) again and examined the apoptosis mechanism in more detail. They reported that aloe emodin applied in a concentration range of 5-30 *μ*M increased the mRNA expression of p53 in both cell lines. An increment in the mRNA levels of p21, Bcl-2, Bax, and cluster of differentiation 95 (CD95) was observed 12 hours after administration of aloe emodin in SJ-N-KP cells, but no transactivation was observed in SK-N-BE(2c) cells. The loss of p53 function in the mutant SK-N-BE(2c) cell line indicates that the results are consistent [69].

**Figure 4:**
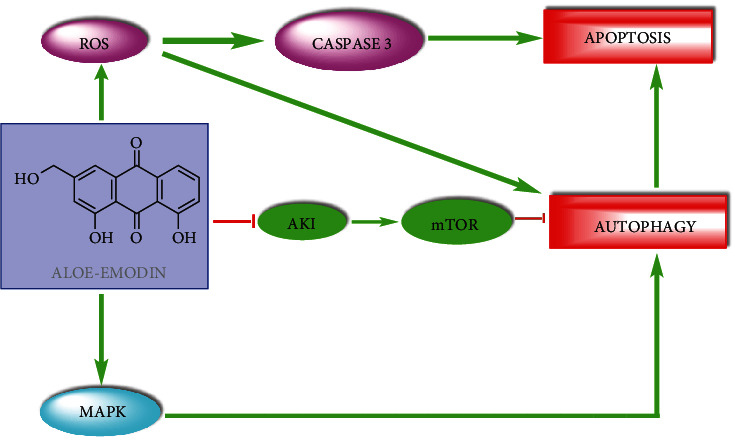
Aloe emodin-induced apoptosis and autophagy (adapted from reference [70]).

The effect of aloe emodin on C6 glioma cells was not related to the cell necrosis due to the lack of a significant increase in lactate dehydrogenase (LDH). Chromatin condensation, one of the characteristic morphological features of apoptosis, was clearly seen in cells administered with aloe emodin, stained with propidium iodide. The toxicity of aloe emodin was partially blocked by the addition of the pan-caspase inhibitor VAD-fm to the experiments; this means that apoptosis is not responsible solely for the antiglioma effect. Later to evaluate the autophagy, acidic vesicular organelles were examined by acridine orange supravitally staining, and by 50-60% bright red fluorescent acidic vesicles were surveyed. In addition, phosphorylated and active forms of mitogen-activated protein kinases (MAPKs), p38, and cJun NH(2)-terminal kinase (JNK), and also phosphorylated IkB (NF-*κ*B inhibitory subunit) were not affected with aloe emodin treatment, but the phospho-ERK level was decreased [55]. More recently, Shen et al. reported that aloe emodin caused caspase-dependent apoptosis and autophagy in A549 and NCI-H1299 lung carcinoma cells. Activation of the MAPK signal, inhibition of the Akt/mTOR pathway, and ROS increase induced autophagy (Figure 4) [70].

Aloe emodin-induced apoptosis was demonstrated by DNA fragmentation gel electrophoresis in human promyelocytic leukemia (HL-60) cells. Caspase-3 levels also increased after administration of 10 *μ*M aloe emodin [42]. The apoptotic and necrotic effect of aloe emodin was recently evaluated in the acute promyelocytic leukemia (HL-60), Burkitt's lymphoma (P3HR-1), and chronic myelogenous leukemia (K-562) cell lines. Annexin-V-FITC/PI double-staining assay results were reported as follows: 33.99% apoptosis and 11.99% necrosis on K-562 and 38.85% apoptosis and 16.66% necrosis on P3HR-1 cell lines. It was concluded that the apoptotic mechanism related to aloe emodin in K-562 and P3HR-1 cell lines was caspase-dependent, while the apoptotic effect was very low (0.54%) and not caspase-dependent in the HL 60 cell line [44].

After the administration of aloe emodin, it was observed that the mitochondrial membrane potential decreased in human tongue squamous carcinoma (SCC4) cells, and the release of apoptosis-inducing factor (AIF), procaspase-9, endonuclease G (Endo G), and Cyt c from mitochondria was observed. It was also observed the increased activity of caspase-3, caspase-8, and caspase-9, reduced levels of PARP and Bcl-2, and increased levels of apoptosis induced activating glucose-regulated protein 78 (GRP78) and transcription factor 6*α* (ATF-6*α*); this may indicate that aloe emodin induces apoptosis and involves endoplasmic reticulum (ER) stress and mitochondria [50]. In a different oral squamous carcinoma (SCC15) cell, the apoptosis mechanism is explained by the regulation of the expression of caspase-9 and caspase-3 [35]. Similarly, upregulation of caspase-3 and Bax protein levels and downregulation of Bcl-2 protein levels in oral mucosa cancer (KB) cells have been described that aloe emodin induces apoptosis [34]. Working with the same cell line before, Xiao et al. stated that the anticancer effect in this cell line was not due to apoptosis, but to phase arrest at the G2/M phase and differentiation induction [54]. In NPC nasopharyngeal cancer cell, it was determined that aloe emodin caused apoptosis by activating caspase-3 and also enhanced DNA fragmentation shown with Comet analysis, and this fragmentation was also shown to be inhibited by the caspase-3 inhibitor Ac-DEVD-CMK [51].

Major cell surface molecular protein named Fas/APO1 belonging to tumor necrosis factor (TNF) receptor family mediates apoptosis induced by external ligand. Another factor that acts a substantial role in the apoptosis process is the p53 molecule, which is a tumor suppressor gene. In the study examining these two factors, also other factors associated with apoptosis, Hep G2 (p53-positive) and Hep 3B (p53-deficient) hepatoma cell lines were used. A strong induction in p53 expression was observed in Hep G2 cells. The augmentation in the amount of p21 protein was observed with both 10 and 20 *μ*g/mL concentration of aloe emodin in Hep G2 cells, while it was clearly observed in Hep 3B at a concentration 20 *μ*g/mL. Fas protein expression was also significant in Hep G2 cells, while no significant difference was found in Hep 3B cells compared to control. Increased proapoptotic Bax expression was observed in both two hepatoma cells, but no difference was found in Bcl-2 protein [40]. The apoptotic mechanism in Huh-7, a different hepatic cancer cell, is the fragmentation of chromatin and observation of apoptotic bodies at 100 *μ*M and reduction of the calpain-2, and ubiquitin protein ligase levels at 200 *μ*M concentrations [41]. It has been previously stated that aloe emodin, which induces apoptosis by the mitochondrial pathway in the hepatic stellate cell (t-HSC/Cl-6), has an antifibrotic effect [71]. A similar study was performed in the human bladder cancer cells (T24). Aloe emodin stimulated the p53 expression, p21, and caspase-3 activation in T24. At the same time, while an increase in Fas/APO1 and Bax expression was observed, a decrease in Bcl-2 was reported [52]. In contrast to these studies, Lin et al. reported that aloe emodin induced apoptosis in human pharyngeal squamous cell carcinoma (FaDu), Hep 3B, and osteosarcoma (MG-63) cells by the p53-independent pathway. The cause of aloe emodin-mediated apoptosis has been explained to be reduced stability of CARP mRNAs and induction of ERK and caspase-8-mediated mitochondrial death pathways [53]. Aloe emodin administration in Hep G2 cells provided significant and sustained activation of JNK, a notable stress-sensitive MAPK. Overexpression of wide-type apoptosis signal-regulating kinase 1(ASK1) increases the phosphorylation of JNK cells and has made the cells susceptible to death from aloe emodin [72]. However, its apoptotic effect with similar mechanisms in healthy liver cell lines (HL-7702) confirms that this compound should be used carefully due to its hepatotoxicity. According to the experiment, it caused S and G2/M phase cell cycle arrest in HL-7702 cells, formation of intracellular reactive oxygen species (ROS), and depolarization of mitochondrial membrane potential (*ΔΨ*m). Cleavage of caspase-3, caspase-8, caspase-9, PARP and upregulation of Fas, p53, p21, and Bax/Bcl-2 ratio levels were also examined [73]. The same study group also proved the toxicity of aloe emodin in the hepatic stem cell HepaRG cell line. In this cell line, aloe emodin showed an increasing effect of ROS formation by depolarization of *ΔΨ*m. It has been concluded that risk assessment must be made before use in humans because it exhibits apoptotic effect through a mechanism that includes Fas death and mitochondrial pathways [74].

Aloe emodin-induced apoptosis in the WiDr colon cancer cell line included caspase-9 and caspase-6 activation. Furthermore, it has shown the downregulation of phosphorylated ERK and potentiation of phosphorylation of p38 and SAPK/JNK. To confirm aloe emodin-induced apoptosis and to compare the role of p38 and JNK, specific inhibitors (SB203580 and SP600125) were used. The fact that both inhibitors caused a reduction in cell death caused by aloe emodin confirmed the results of the study [75]. In a previous study, WiDr and DLD-1 colon cancer cells were used. The apoptosis-inducing effects of aloe emodin were the release of AIF and Cyt c from mitochondria, activation of caspase-3, and hence DNA fragmentation and nuclear shrinkage. Also, the activity of casein kinase II (CK2) and the phosphorylation of Bid, a downstream substrate of CK2 and a proapoptotic molecule, were decreased [76].

The cause of aloe emodin-mediated apoptosis in melanoma (A375) cells has been reported as increased activity of caspases and downregulation of nitric oxide synthase (iNOS), with decreased Bcl-2 expression [56]. The apoptosis mechanism in epidermoid (A431) and neck squamous cell (SCC25) carcinoma cells is revealed as upregulation of TNF-*α* and Fas ligand, downstream adaptor TNF-R1-associated death domain and Fas-associated death domain, and activation of caspase-8 [57].

In gastric carcinoma (AGS and NCI-N87) cell lines, treatment of 0.15 mM aloe emodin caused apoptosis by increased fragmented nuclei and the release of AIF and Cyt c. Caspase-3 activity was significantly induced in AGS cells 24 hours after aloe emodin administration and in NCI-N87 cells after 48 hours [77]. In the SGC-7901 gastric cancer cells, Lin et al. reported that application of 10 *μ*M aloe emodin and 12.8 J/cm^2^ illuminating energy enhanced the levels of caspase-3 and caspase-9 proteins. They explained that the mitochondrial pathway may be involved in apoptosis [78].

**Figure 5:**
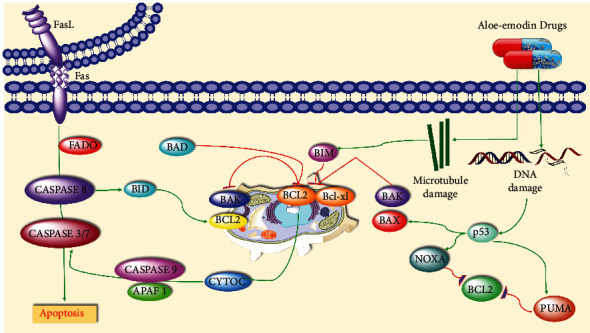
Aloe emodin-induced apoptosis (adapted from reference [83]).

Guo et al. stated that the effect of aloe emodin at a concentration of 2.5, 5, 10, 20, and 40 *μ*mol/L on growth inhibition in human cervical cancer (HeLa) cells was due to cell cycle arrest, not apoptosis induction [38]. It has been explained that the apoptosis mechanism of aloe emodin in the breast cancer (MCF7) cell line is related to mitochondrial and endoplasmic reticulum death pathways [30]. In a different study, the apoptosis mechanism in the MCF-7 cell line was explained with increased Fas expression [79]. In a study comparing tamoxifen and aloe emodin on MCF7 cells, apoptosis was found to be 30.2% and 29.3%, respectively. Aloe emodin application has a similar effect to tamoxifen, and the insulin-like growth factor type 1 receptor (IGF-1R), insulin-like growth factor-binding protein 2 (IGFBP-2), and B-raf gene expressions were downregulated [80]. In SkBr3 breast cancer, aloe emodin increased cleaved PARP and exhibited an apoptotic effect by suppressing the PI3K/Akt/mTOR signal transduction pathway [81]. The summary of the studies in which the mechanism of apoptosis induced by aloe emodin was elucidated is given in Table 2 and illustrated in Figure 5.

### 4.3. Antimetastatic and Antiangiogenic Activities of Aloe Emodin

The high cancer-related death rates result from the metastatic spread of tumor cells from the primary site. Angiogenesis, cell invasion, cell adhesion, and cell proliferation are interrelated biological events essential for tumor metastasis. The role of aloe emodin in the inhibition of PKC isozymes, ERKs, and p38 is an indication that it may play a critical role in the progress of cancer metastasis [60]. The effects of aloe emodin on migration, invasion, adhesion, and FAK (focal adhesion kinase) expression were investigated with the study performed in HO-8910PM ovarian cancer cell line. Results clearly demonstrate that 20 to 80 *μ*mol/L concentrated aloe emodin decreased the migrated cell number and adhesion to Martigel compared with the control group. The invasive ability of the cells also decreased between the ranges of 10.2-46.5%. Inhibition of FAK expression levels associated with the invasive potential of tumors is one of the targets of anticancer drugs, and aloe emodin has been found to downregulate the mRNA expression of FAK [84].

The clear sign of the disruption in the migration mechanism with the application of aloe emodin in B16-F10 melanoma cells is the star-dendritic cell morphology and the rearrangement of actin fibers. Aggregation, migration, adhesion, and invasion data also confirmed the results. Cell adhesiveness and stiffening were increased after aloe emodin treatment and related to the participation of the transamidation form of TG2-dependent (transglutaminase 2) formation of extracellular matrix (ECM) protein cross-links [85]. The later study of Tabolacci et al. in human melanoma cells (A375 and SK-MEL-28) confirms the results in murine melanoma cells. An increase in the transamidation activity of the transglutaminase, whose expression did not change, and hence cell differentiation were detected significantly. It has been stated that 30 *μ*M aloe emodin therapy causes significant inhibition in the proliferation, stemness, and invasion of the melanospheres [86]. In a different research, invasion inhibition of nasopharyngeal cancer cells (NPC) by 40 *μ*M aloe emodin treatment which causes a decrease inMMP-2 expression *via* the p38 MAPK-NF-*κ*B signaling pathway has also been reported [76].

In the study investigating the effect of aloe emodin (25–50 *μ*M) on matrix metalloproteinase-9 (MMP-9), which is associated with tumor invasion, migration, and metastasis in cancer cells, Chen et al. stated that MMP9 gene expression was inhibited in SCC-4 cells. The protein level and activity of MMP-2 were also inhibited, but gene expression was not affected. Aloe emodin has been disclosed to inhibit the migration and invasion of tongue cancer cells [87]. Liu et al. demonstrated that aloe emodin (0–15 *μ*M) inhibited the proliferation, knocking Rictor which is a unique binding partner of rapamycin complex 2 (mTORC2) and plays an important role in cell proliferation and tumor metastasis, in PC3 cells and disrupting anchorage-independent colony formation [39].

Epithelial-mesenchymal transition (EMT) is a significant factor in cancer metastasis. Increasing the expression of Y-box binding protein 1 (YB-1), which plays a role in transcription and translation, supports cell proliferation and inhibits apoptosis, tumor invasion, metastasis, and angiogenesis. It has been found that aloe emodin (10-40 *μ*M) can suppress expression of YB-1 in breast cancer cells overexpressing HER-2 and suppress cancer metastasis and cancer stem cells by inhibiting EMT [81]. It has also been shown to inhibit invasion and adhesion in the breast cancer cell line (MDA-MB-231). Aloe emodin suppressed metastasis by inhibiting invasion by 52.98%, fibronectin and laminated adhesion by 34.99% and 28.73% at 80 *μ*mol/L concentration [88]. Adhesion, migration, and invasion inhibiting effect of aloe emodin (5 to 20 *μ*M) photodynamic therapy in MCF-7 breast cancer cell line was shown in Chen et al.'s research. MMP2, MMP9, nuclear factor erythroid 2-related factor 2 (Nrf2), and vascular endothelial growth factor (VEGF) expressions clearly indicated that induction of metastasis was linked to oxidative stress [30]. In Haris et al.'s research, the enhanced expression of the metastasis suppressor 1-like gene (MTSS1L) showed an antimetastatic effect of 58.6 *μ*g/mL concentrated treatment of aloe emodin in U87 glioblastoma cell line [89].

In Hep G2 cell line, 10-40 *μ*M aloe emodin upregulated the metastasis suppressor nucleoside diphosphate kinase A (nm23) in a dose-dependent manner [72]. In colon cancer cell line (WiDr), mRNA expression, and promoter/gelatinolytic activity of MMP-2/9, RhoB expression was downregulated by aloe emodin (10-40 *μ*M) treatment. In the same study, it was shown that 20 *μ*M concentrated aloe emodin prevents migration, invasion, and tube formation in endothelial cells (HUVECs) in order to prove its antiangiogenic effect [75]. Chen et al. investigated the combined effect of aloe emodin-photodynamic therapy in the HUVECs cell line. The combination therapy inhibited the migration and invasion. An increase in p38 and Perk expressions, a decrease in VEGF, and no change in pJNK expression were observed after treatment. The cellular antimetastatic effect of aloe emodin has been associated with MAPK signaling pathway [90]. The antiangiogenic effect of aloe emodin on aortic endothelial cells (BAEC) has been extensively studied. In the cell line treated with aloe emodin, cell proliferation, tube formation by endothelial cells in Matrigel, zymographic analysis for detection of MMP2/9, and urokinase and fluorescent cell migration and invasion analysis were performed. In addition, an *in vivo* chicken chorioallantoic membrane (CAM) test was also performed. Aloe emodin performed *in vivo* angiogenesis inhibition in 60% of the treated eggs at 50 nmol concentration and at 30 nmol concentration, and it was found with 50% inhibition. Application of 25 *μ*M aloe emodin weakly inhibited endothelial cell migration, and no effect on tumor cell (HT-1080) migration was observed. Contrary to what was previously reported in other studies, it was stated in this study that aloe emodin had no anti-invasive effect. While there was a dose-dependent increase in MMP-2 levels in BAEC, MMP-2 levels decreased in the HT-1080 cell line, and no important effect on MMP-9 levels was observed. A dose-dependent decrease in urokinase levels was detected in both the HT-1080 and BAEC cell lines. HUVEC tubule formation was completely inhibited by 25 *μ*M aloe emodin. According to the results of the study, the antiangiogenic effect of aloe emodin was attributed to its potent urokinase and tubule formation inhibitory effect [91]. In a study evaluating the effects on retinal neovascularization due to hypoxia in a VEGF secretion model, aloe emodin inhibited the vascular endothelial growth factor A (VEGFA) secretion, decreased the mRNA expressions of VEGFA and PHD-2 (prolyl hydroxylase-2) in human retinal pigment epithelial cell line (ARPE-19). It has been reported that the inhibitory mechanism of action against hypoxia-induced retinal neovascularization is due to inhibition of the HIF-1*α*/VEGF signaling pathway [92].

## 5. Potential of Synthesized Derivatives of Aloe Emodin

**Figure 6:**
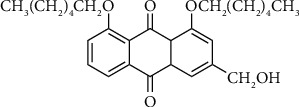
Chemical structure of 1,8-di-O-hexylaloe-emodin.

By the reason of significant anticancer activity, aloe emodin has attracted the attention of synthetic organic chemistry in order to obtain the more active components. Several studies have revealed many different derivatives to have been synthesized which display activity in varying degrees. Aloe emodin synthetic derivatives prepared by alkylation of aliphatic hydroxyl group and the substitution with different groups such as amino, thiocyano, and selenocyano exhibit higher cytotoxic activity than aloe emodin. Among alkyl derivatives including 1,8-di-O-alkylaloe-emodins, 15-aminochrysophanols, 15-amino-1,8-di-O-hexylchrysophanols, 15-thiocyano-, and 15-selenocyanochrysophanols as well as 1,8-di-O-hexyl-15-thicyano- and 1,8-di-O-hexyl-15-thicyanochrysophanols, the remarkable cytotoxic effect on HCT 116 cells was determined by treatment with 1,8-di-O-hexylaloe-emodin (Figure 6). Diethylamino, pyrrolidinyl, piperidinyl, 4-methylpiperazinyl, and imidazolyl substitution of the aloe emodin induce 4.6-2.4-fold increase on cytotoxic activity against HCT 116 cells. While modification of hydroxymethyl substituent with diethylamino, 4-methylpiperazinyl, and imidazolyl groups increased the cytotoxic activity on HCT 116 cells, pyrrolidinyl and piperidinyl groups which lead to insignificant changes as compared to aloe emodin. The modification of hydroxyl group with SeCN and SCN groups also increased the cytotoxic effect. On the other hand, all amino derivatives enhanced the effects on stably P-gp-expressing Hep G2 cells. Selenocyano derivatives have higher cytotoxic effect on stably P-gp-expressing Hep G2 cells than the corresponding thiocyano derivatives. 15-Aminochrysophanols, 15-amino-1,8-di-O-hexylchrysophanols, 15-selenocyanochryso phanol, 1,8-di-O-hexyl-15-thiocyanochrysophanol, and 1,8-di-O-hexyl-15-selenocyano chrysophanol displayed notable cytotoxic effects on stably P-gp-expressing Hep G2 cancer cells [93].

**Figure 7:**
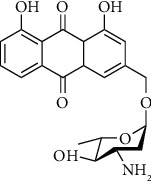
Chemical structure of amino-sugar derivative of aloe emodin.

Aloe emodin glycosides carrying amino-sugar substitution have also been synthesized in order to increase potential anticancer activity. Doxorubicin, which is an antineoplastic agent used in the management of hematopoietic and solid tumors clinically, has been classified as anthracycline and has the same core structure with aloe emodin substituted amino-sugar unit. Resistance to doxorubicin is the essential problem, which restricts the usage and leads to the further research on discovery of new chemotherapeutic agents. Therefore, Breiner-Goldstein et al. designed and synthesized aloe emodin amino-sugar derivatives. Significant improvements in cytotoxic activity were observed by synthesized derivatives against several cancer cell lines such as leukemia, ovarian, and breast cancer lines, which have doxorubicin resistance in different levels. Amino-sugar derivatives (Figure 7) were designed in four different combinations of two structures following the configurations of the glycosidic linkage in *α* or *β* and the carbohydrate C-3 amine in axial or equatorial position. Notable differences among the cytotoxic activities of synthesized derivatives have been determined in all of the tested cell lines, and the derivative containing an *α*-glycosidic linkage and an equatorial carbohydrate C-3 amine displayed the most potent cytotoxic activity [94].

**Figure 8:**
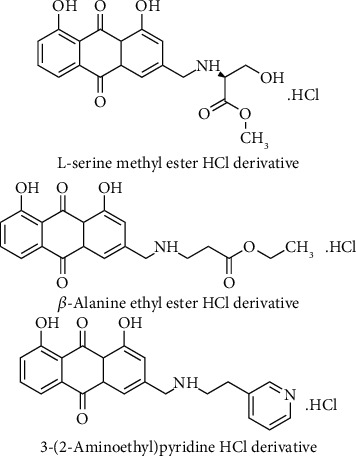
Chemical structures of L-serine methyl ester HCl, *β*-alanine ethyl ester HCl, and 3-(2-Aminoethyl)pyridine HCl derivative of aloe emodin.

Synthetic derivatives of aloe emodin to increase solubility in water by bonding with different amino acid esters and substituted aromatic amines were prepared and investigated for their antitumoral effects. Transformation of hydroxymethyl group of aloe emodin that placed position 3 leads to the occurrence of compound 3-(bromomethyl)-1,8-dihydroxyanthracene-9,10-dione treatment with amino acid methyl esters and amino acid ethyl esters, and substituted aromatic amines resulted to the corresponding alkylamino derivatives. Aloe emodin derivatives exhibited significant cell growth inhibition on NCI-H460 (human lung cancer cells) and Hep G2 (human liver cancer cells), and some of the derivatives display comparable antitumor activity against HeLa (human epithelial carcinoma cells) and PC3 (prostate cancer cells), with aloe emodin. Among the synthesized derivatives compounds containing *β*-alanine ethyl ester, L-serine methyl ester, and 3-(2-aminoethyl) pyridine substituents which were established as (S)-methyl 2-(((4,5-dihydroxy-9,10-dioxo-9,10-dihydroanthracen-2-yl) methyl) amino)-3-hydroxypropanoate hydrochloride, 1,8-dihydroxy-3-(((2-(pyridin-3-yl) ethyl) amino) methyl) anthracene-9,10-dione hydrochloride and ethyl 3-(((4,5-dihydroxy-9,10-dioxo-9,10-dihydroanthracen-2-yl) methyl) amino) propanoate hydrochloride displayed higher antitumor activity against NCI-H460 and Hep G2 tumor cell lines than aloe emodin. The structure-activity relationship study displayed that *β*-alanine ethyl ester, L-serine methyl ester, and 3-(2-aminoethyl) pyridine substituents (Figure 8) were considered important for antitumor activity. The existence of oxygen atoms in high amount and hydroxyl group of aliphatic esters in *β*-alanine ethyl ester and L-serine methyl ester derivatives increased the water solubility by hydrogen bonding and probably leads to improvement in cell permeability. Therefore, these groups were suggested responsible for enhanced antitumor activity [95].

**Figure 9:**
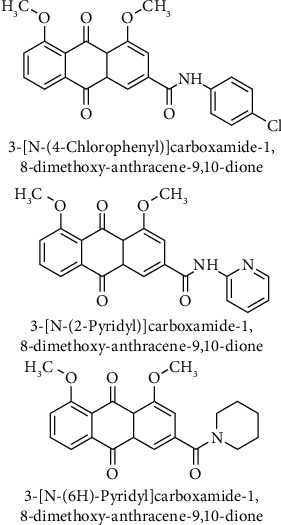
Chemical structures of 4-chlorophenyl-, 2-pyridyl-, and (6H)-pyridyl-carboxamide-1,8-dimethoxyanthracene-9,10-dione.

Aloe emodin has some disadvantages for oral administration due to its poor intestinal absorption, fast elimination, and bioavailability *in vivo*. A series of compounds synthesized by transforming methylol at the position 15 to amide. 4-Chlorophenyl, 4-methylphenyl, 2-pyridyl, (6H)-pyridyl, (4H)-pyrrolyl morpholinyl, and 1-naphthyl derivatives of 3-carboxy-1,8-dimethoxyanthracene-9,10-dione (Figure 9) were designed and synthesized to evaluate inhibitory activity on proliferation of human non-small cell lung carcinoma (A549), human breast adenocarcinoma (MDA-MB-231), and human cervix carcinoma (HeLa) cells. Higher antiproliferative activity was observed with 4-chlorophenyl, 2-pyridyl, and (6H)-pyridyl derivatives than aloe emodin for MDA-MB-231 cells at a concentration of 3 *μ*M, and only 4-chlorophenyl and (6H)-pyridyl derivatives displayed better antiproliferative activity against A549 cells at the same concentration level. The anticancer activities of the aloe emodin derivatives were determined as only slightly higher than that of aloe emodin. Modification of 15-hydroxymethyl to amide did not result in significant changes as expected [96].

**Figure 10:**
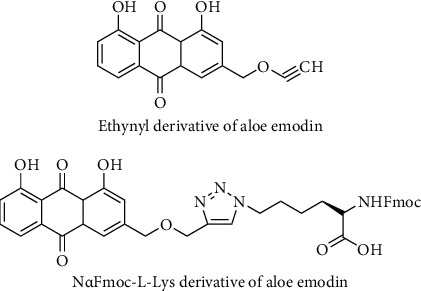
Chemical structures of ethynyl and N*α*Fmoc-L-Lys derivatives of aloe emodin.

Semisynthetic anthraquinone derivatives, which were prepared with the N*α*Fmoc-L-Lys and ethynyl group from aloe emodin, were synthesized (Figure 10). Aloe emodin derivative containing N*α*Fmoc-L-Lys group displayed significant anticancer activity against human cervix cancer (HeLa), and this activity was found to be comparable with anticancer agent 5-FU used as reference at the same concentration. Similar results were observed against human colon cancer (HT-29) and human prostate cancer (PC-3) cell lines for the same derivative while the derivative containing ethynyl substituent did not show inhibition against human prostate cancer (PC-3) cell lines. Modified derivatives of emodin, which were prepared in the same way, were also determined inactive in the same study. It does not have a methyl group (–CH_3_) at position 6 and carries a methylene at position 3. Substitution with different functional groups such as alkyls, hydroxyls, hydroxyl group of aliphatic esters, alkyl amines, ammonium salts, and halogens in dissimilar location of the skeleton of aloe emodin was reported to have great importance for anticancer activity which was revealed by structure-activity relationship (SAR) studies [97].

**Figure 11:**
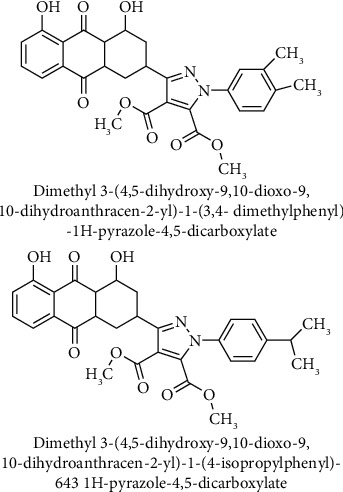
Chemical structures of pyrazole-linked aloe emodin derivatives.

Recently, Kumar et al. designed and synthesized pyrazole-linked aloe emodin derivatives to improve potential anticancer activity of aloe emodin. Free hydroxyl group placed at the C-1 and C-8 positions of aloe emodin was found to be essential for cytotoxic activity. Therefore, pyrazole derivatives of aloe emodin were prepared by modification of C-3 position containing CH_2_OH group. Among all synthesized derivatives, remarkable inhibitory activities were observed with dimethyl 3-(4,5-dihydroxy-9,10-dioxo-9,10-dihydroanthracen-2-yl)-1-(3,4-dimethylphenyl)-1H-pyrazole-4,5-dicarboxylate and dimethyl 3-(4,5-dihydroxy-9,10-dioxo-9,10-dihydroanthracen-2-yl)-1-(4-isopropylphenyl)-1H-pyrazole-4,5-dicarboxylate (Figure 11) against MDA-MB-231 breast cancer cells in a concentration-dependent manner. It has also been reported that both aloe emodin derivatives induce apoptosis in early and late phases and arrest the cell cycle at the G2/M phase in MDA-MB-231 cells [98].

Aloe emodin has little therapeutic activities with all other free anthraquinones, due to its poor intestinal absorption, short elimination half-life, and low bioavailability. Therefore, usage of aloe emodin is limited clinically. By this reason, several derivatives have been synthesized in order to improve bioavailability and activity of the compound. Many studies have revealed that new derivatives of aloe emodin were synthesized, and some of them exhibited significant cytotoxic activity. However, further preclinical as well as clinical studies are needed to clarify the activity of these synthesized derivatives.

## 6. Nanocarrier-Based Application of Aloe Emodin

Natural molecules, like anthraquinones, have displayed promising anticancer properties in studies. Despite the achieved important results, poor solubility, narrow therapeutic window, low absorption rate, and low bioavailability, stability problems limit the use of molecules [99, 100]. Also reported are the properties of molecules with an unfavorable pharmacokinetic profile and a reduced elimination half-life that needs to be improved [101].

Phytochemicals are compounds that have shown important results in anticancer therapy in recent years [101]. One of them is aloe emodin, which is the primary component of anthraquinones and exhibits antiproliferative properties on a variety of tumor cells and alters the expression of a number of proteins involved in oxidative stress, antimetastasis, and apoptosis [57, 102]. Although it is shown as a promising antitumor candidate in the literature due to its important activity against various tumors such as breast, lung, and liver cancers, the use of aloe emodin and its pharmaceutical application are limited due to its rapid degradation and poor bioavailability [103–105]. In general, research has focused on elucidating the molecular mechanism of action. At this point, new drug release systems developed with aloe emodin become remarkable.

Colloidal nanoscale drug delivery systems such as nanoparticles, nanostructured lipid carriers, solid lipid nanoparticles (SLN), and nanoliposomes are effective formulations and considered suitable for solid tumors therapies to overcome the drawbacks mentioned above, to protect the therapeutics from degradation, and to release the active molecule in a controlled fashion as well as can be targeted providing a great advantage in cancer treatment [106, 107]. It is essential for the success of treatment that chemotherapy drugs target tumors as much as probable and have a limited effect on healthy tissues [108]. Nanopharmaceutics come into play here, especially with the targeted carrier systems; the drug cannot reach healthy tissues from the bloodstream since the drug is not free. This point opened important horizons and was expressed as a promising situation. It has been reported that nanodrug delivery strategies of natural anthraquinone equivalents such as alchemix, aloe emodin, emodin, and many synthetic analogues are progressive tools that transport drugs to tumor cells with minimal drug leakage into normal cells [109].

In recent years, FDA-approved targeted nanodrugs have attracted attention in targeted cancer treatment. It minimizes unwanted side effects by delivering chemotherapeutic agents to cancer cells [110]. In the literature, phytochemicals are potential anticancer agents, but when used, it is stated that certain difficulties must be overcome in order to achieve the maximum effect. There are limited sources of aloe emodin, and the use of nanocarriers developed with many phytochemicals belonging to different classes is a new way, but progressing to clinical trials from *in vitro* studies is available [107].

One of the limited studies conducted to improve the physicochemical and pharmacokinetic properties and increase the anticancer effect by overcoming the limits with nanocarrier systems was carried out by Wu et al., poly(lactic-co-glycolic acid) nanoparticle formulation prepared to increase its effectiveness on human lung squamous cell carcinoma. In the study, compared to unloaded aloe emodin, significant suppression of cancer cell proliferation, induced cell cycle arrest, and apoptosis were reported with nanoformulation. *In vivo*, aloe emodin nanoparticles had an inhibitory effect on tumor growth with little toxicity [111].

Freag et al. investigated the novel injectable surface-functionalized polyethylene glycol (PEG) liquid crystalline nanoparticles to improve its water solubility and enhance its anticancer effect on breast cancer cells with *in vivo* and *in vitro* studies. Nanoparticles with submicron particle size and approximately 96% drug encapsulation efficiency reduced the monoolein hemolytic tendency to 3% with PEGylation approach, which modifies the surface of nanocarriers, extends their circulatory half-life, promotes their accumulation in tumors owing to its enhanced permeability and retention effect (EPR effect), and increases the serum stability of the nanoparticles. Increased cellular uptake was observed in MCF-7 cells, and PEGylated aloe emodin LCNPs showed an important increase in bioavailability compared to free aloe emodin *in vivo* assessment [112]. The steric stable structure formation provided by the PEGylation process, the plasma circulation of aloe emodin PEGylated nanoparticles has increased compared to nanoparticles, and it has become possible to be targeted significantly in cancer with a decrease in biodistribution. Despite these achieved results, it is thought that the anticancer effect should be evaluated with *in vivo* studies mostly in animal cancer models.

In another study, aloe emodin-loaded SLN were developed and characterized with particle size, drug entrapment efficiency, zeta-potential, and stability. Their cytotoxicity was assessed through different cell lines such as human breast cancer MCF-7 cells and human hepatoma Hep G2 cells compared to the aloe emodin solution, and it was found that while toxicity was not observed on normal human breast epithelial MCF-10A cells, it was seen on cancer cell lines due to the increased cellular uptake of SLN compared to aloe emodin solution which is attributed to the increased cellular uptake of SLN compared to aloe emodin solution [103]. Based on these findings, it can be an effective way to improve anticancer efficacy, but since this study was performed only in *in vitro* models, it is thought that it is necessary to reach comprehensive results by conducting *in vivo* studies.

Besides surgery and chemotherapy, which are the traditional treatment methods in the treatment of gastric carcinoma, which has high incidence and mortality rates in China, photodynamic therapy has been introduced as a new management process in current experimental studies, which may be in the fight against cancer. In one study, the efficiency of the combination of photodynamic therapy and gene transfection on gastric carcinoma was investigated using aloe emodin-encapsulated nanoliposomes, which are effective carrier systems for gene therapy as well as a photosensitizer. Photosensitizer structures selectively accumulate in tumor tissues, which play a critical role in photodynamic therapy and the anticancer effect of aloe emodin that is known as one of the photosensitizers. A single reactive oxygen species (ROS) occurs in photochemical reactions causing photodamage to tumor cells. In this study, the research group detected the expression of the caspase-3 after transfection, which is a protein enzyme that plays a critical role in apoptosis. In addition to transfecting, the recombinant plasmid by preparing aloe emodin loaded nanoliposomes; it was aimed at increasing the bioavailability of aloe emodin. It was found that higher protein expression of the caspase-3 than controls and mortality increased to 77.3% at 12 hours in transfected cells [104]. It can be said that the apoptotic rate in human gastric cancer cells can be significantly increased by these improved aloe emodin nanoliposomes.

Liposome formulation, which is one of the ways to increase skin penetration, has increased the passage of many molecules due to its similarity to cell membrane structure [113]. Chou and Liang assessed skin permeability profiles and molecular effect and cytotoxicity of aloe emodin developed with the liposomal formulation. A specific dose-dependent cytotoxic effect of aloe emodin on human epidermoid carcinoma A431 cells and human neck SCC SCC25 and head cells and the inhibited skin cancer cell proliferation were observed, and cell death was accelerated in a short time with the application of liposomal aloe emodin. Also, this liposomal formulation increased the ability of aloe emodin to penetrate the skin [57].

The use of drug delivery carriers developed with inorganic nanomaterials has increased to improve bioavailability for drugs with poor water solubility, which limits their use in recent years. One of them is the nanoscale mesoporous silica particles, especially SBA-15, which is the most preferred drug carrier due to its advantages. Nanoscale SBA-15 particles can easily enter the cell through endocytosis; therefore, it can be considered an emerging option for the delivery and targeting of aloe emodin to cancer cells. Jangra et al. aimed to increase the bioavailability of aloe emodin extracted from leaves of *Aloe vera* with SBA-15 particles and to examine its *in vitro* release properties and cytotoxic effect on HeLa cell line. It was found that these nanoscale SBA-15 particles showed significant potential for antineoplastic effect by destroying the nucleus of cancer cells [114]. It can be said that SBA-15, which clinically increases the effectiveness of anticancer drugs such as aloe emodin, is a new alternative option that can be applied. It is a common result that nanobased carriers when considered together with other studies can increase the efficacy of aloe emodin in clinical applications.

## 7. Toxicity of Aloe Emodin

Some anthraquinones have been found to be mutagenic mostly in *in vitro* assay systems. Early studies have shown that danthron and aloe emodin cause significant increases in various revertant colonies in *Salmonella* strains (TA1537, TA1538, and TA98) [115, 116]. Again, in different studies conducted in the 1990s, it has been proven that emodin, danthron, and aloe emodin at micromolar concentrations increase the formation of micronuclei in mouse lymphoma (L5178Y) cells dose-dependently and cause moderately increased mutant frequencies. Aloe emodin was also determined to cause DNA strand breaks in the comet test at a concentration of 50 *μ*M. The genotoxic and mutagenic properties of these studied anthraquinones are based on the inhibition of the catalytic activity of topoisomerase II [117, 118].

In the early 2000s, exposure of human skin fibroblasts to aloe emodin and ultraviolet radiation was shown to cause significant phototoxicity [119]. In recent years, articles have been published proving that aloe emodin causes hepatotoxicity and nephrotoxicity. In a study with methanol extracts of *C. occidentalis* seeds, it was reported that the extracts had a pronounced toxic profile in rat primary hepatocytes and Hep G2 cells, and aloe emodin may be one of the main components responsible for toxicity [120]. Similarly, *in vivo* studies with *Rheum palmatum* have shown that aloe emodin may be the primary secondary metabolite responsible for hepatic and renal toxicity [121]. In a study conducted in 2019, it was revealed through the mechanisms that Aloe emodin significantly inhibits liver development in zebrafish and reduces the expression of liver-type fatty acid binding protein. The mechanism elucidated was primarily stated as the activation of the NF-*κ*B-P53 inflammation-apoptosis pathway, but no effect on IL-6 and JAK3 was observed [122]. Evidence that aloe emodin induces primary damage to DNA in the liver and kidneys of mice in *in vivo* comet analysis has also been associated with its being hepatotoxic and nephrotoxic [123]. In addition to all these, the information that aloe emodin is genotoxic was emphasized in the EFSA panel [124]. It turns out that studies elucidating the toxic effects of aloe emodin for clinical use are insufficient and risk assessment of aloe emodin exposure is necessary.

## 8. Conclusion

In current years, with the increasing incidence of cancer, several studies have been carried out on the anticancer effect of bioactive compounds. Natural compounds extracted from various food and plants might provide likely alternatives to standard anticancer agents. It is well known that anthraquinones have anticancer activities against various cancer types.

This review summarizes the studies describing the anticancer activity of aloe emodin, available in the recent years, either alone or as a main constituent of plant extracts. Based on literature data, it can be concluded that aloe emodin is one of the major components of the extracts obtained from several medicinal plants.

As an outstanding antineoplastic agent, aloe emodin has wide application possibilities, confirmed by numerous pharmacological data. Studies focusing on aloe emodin have demonstrated its antitumor mechanism, but its toxicity, pharmacokinetics, and pharmaceutical properties are generally not emphasized. Although there is limited data on its toxicity, it is clear that more research is required.

Studies have shown that the pharmacological efficacy of liposomal aloe emodin is deeper against nonencapsulated aloe emodin and that aloe emodin can be formulated into nanoparticulate drug delivery systems to increase the distribution of the active constituent.

In conclusion, aloe emodin displays great potential as an antineoplastic agent with possible use as a cytoprotective and/or synergistic agent as part of conventional cancer management. Several *in vitro* results support this claim, yet more research is necessary to clarify the molecular mechanisms *in vivo*, along with exploring its possible use as a prophylactic agent clinically. Aloe emodin may eventually show to be another phytonutrient with anticancer properties. It could be recommended the continuing focus on clinical trials and toxicological studies on a range of cancer, targeting to appraise the efficacy of aloe emodin, together, alone, and as a constituent of plant extracts, and to define an efficient and safe dose.

## Figures and Tables

**Table 1:** The effects of aloe emodin on cell cycle.

Cancer type	Cell line	Dose	Cell cycle arrest	Mechanism	Reference
*Colon cancer cell line*	WiDr	30 *μ*mol/L/48 h	Cell cycle distribution: G0 (2%), G1 (20.3%), S (12%), and G2/M (64.2%)	Inhibition of cyclin B1 promoter	[49]
*Esophageal cancer cell lines*	TE1	0, 2.5, 5, 10, and 20 *μ*M/48 h	The number of cells in the S phase decreased depending on the dose	Inhibition of cyclin D1 transcription activity	[36]
*Human malignant glioma cells*	U87	58.6 *μ*g/mL/24 and 48 h	Induced S phase arrest	A decrease in mitochondrial membrane potential (*ΔΨ*m)	[24]
*Cervical cancer cell line*	HeLa	2.5, 5, 10, 20, and 40 *μ*mol/L/72 h	Increased distribution of cells in the G2/M phase	A decrease in cyclin A and (CDK2), increase in cyclin B1 and CDK1	[38]
*Tongue squamous cancer cell line*	SCC-4	10, 20, 30, 40, and 50 *μ*M/48 h	S phase arrest	Increase in p53, p21, p27 Decrease in cyclin A, E, thymidylate synthase, and Cdc25A (cell division cycle 25 A)	[50]
*Nasopharyngeal carcinoma cell line*	NPC	60 *μ*M/24 and 48 h	G(2)/M phase arrest	Increase in cyclin B1 level	[51]
*Hepatoma cell line*	Hep G2	10 and 20 *μ*g/mL/24 and 48 h	Cell accumulation in the G1 phase	p53 accumulation and increase in CDK inhibitor expression	[40]
*Human bladder cancer cell line*	T24	5,10,25, and 50 *μ*M/24 h	G2/M phase arrest	Inhibition of cyclin B1 expression and increase in p53 expression	[52]
*Pharyngeal squamous cell carcinoma line* *Lung cancer cell line* *Hepatoma cell line*	FaDu H1299 Hep 3B	60 *μ*M/24 and 48 h	S phase arrest	Increase in cyclin A and E2F1 (E2F transcription factor 1)expression, and CDK2 phosphorylation	[53]
*Oral cancer cell line*	KB	10 to 40 *μ*M/1, 2, 3, 4, or 5 days	G2/M phase arrest	KB cells have the wild-type p53 gene and have been reported to be sensitive to aloe emodin	[54]
*Glioma cell line*	U251	20 *μ*M/24 h	G2/M phase blocking	A decrease in the active form of ERK1/2 (the extracellular signal-regulated kinase ½)	[55]
*Promyelocytic leukemia cell line*	HL-60	5,10,15, 20, and 25 *μ*M/48 h	G2/M arrest	Increase in CDK1 activity and p27 levels	[42]
*Human glioma cell line* *Transformed glia cell line*	U-373MG SVG	40 *μ*M/48 h	S phase arrest	Decreased protein kinase C (PKC) activity	[26]
*Gastric cancer cell line*	MKN45	0.05 Mm/24 and 48 h	G0/G1 phase arrest	A decrease in the spermine levels	[33]
*Gastric cancer cell line*	SGC-7901	2.5, 5, 10, 20, and 40 *μ*M/48 and 72 h	G2/M phase arrest	A decrease in cyclin A and CDK2, increase in cyclin B1 and CDK1 expressions	[31]
*Murine melanoma*	*B16*	40 *μ*M/24 h	G2/M phase arrest	p53 accumulation and increased expression of cyclin D1 and D3	[56]
*Skin cancer cell lines*	A431 SCC25	25.9 *μ*M(A4319 and 19.3 (SCC25) *μ*M/24 and 48 h	S-G2/M phase arrest	Upregulation of p53	[57]
*Breast carcinoma cell line*	SkBr3	20 and 40 *μ*M/24, 48, and 72 h	Sub-G1 cell cycle arrest	Cleavage of PARP (poly(ADP-ribose) polymerase)	[58]

**Table 2:** Induction of apoptosis by aloe emodin.

Cell line	Concentration	Apoptosis mechanism	Reference
*CH27* *H460*	40 *μ*M	Caspase-3 activation, increase in Cyt c Changes in PKC isozymes	[61]
*CH27*	40 *μ*M	Modulation of the expression of Bcl-2	[62]
*H460*	40 *μ*M	Nucleophosmin release to the cytosol, nucleophosmin degradation	[63]
*H460*	40 *μ*M	DNA aggregation	[64]
*H460*	40 *μ*M	Protein kinase, Bcl-2, caspase-3, and p38 protein expression modulation	[65]
*H460*	40 *μ*M	Increased expression of HSP70, 150 kDa oxygen-regulated protein, protein disulfide isomerase, and HSP60	[66]
*H460*	20 *μ*M	Reduction in Bcl-2 protein levels and proform caspase-3 and caspase-7 protein expression levels Increase in proform of caspase-8 and caspase-9	[67]
*U-373MG*	40 *μ*M	Decreased PKC isozyme levels, induction of caspase-7 activation	[26]
*U87*	58.6 mg/mL	Collapse in *ΔΨ*m, oligonucleosomal fragmentation	[24]
*SJ-N-KP*	25 *μ*M	Cell shrinkage, membrane bubbles, and nuclear fragmentation	[25]
*C6*	20 *μ*M	Increase in LDH, chromatin condensation, decreased phospho-ERK level	[55]
*HL-60*	10 *μ*M	Increased caspase-3 levels, DNA fragmentation	[42]
*SCC4*	30 *μ*M	Increased the release of AIF, procaspase-9, Endo G, and Cyt c Caspase-3, caspase-8, and caspase-9 activation Decrease in *ΔΨ*m, PARP, and Bcl-2 levels	[50]
*SCC15*	50 *μ*M	Regulation of the expression of caspase-3 and caspase-9	[35]
*KB*	40 *μ*mol/L	Upregulation of caspase-3 and Bax protein Downregulation of Bcl-2 protein	[34]
*NPC*	60 *μ*M	Caspase-3 activation, DNA fragmentation	[51]
*Hep G2* *Hep 3B*	10 and 20 *μ*g/mL	Increased p53 expression (Hep G2) Increased p21 protein level, proapoptotic Bax expression (Hep G2 and Hep 3B)	[40]
*Huh-7*	100 *μ*M and 200 *μ*M	Chromatin fragmentation, decreased calpain-2, and ubiquitin protein ligase levels	[41]
*T24*	5 to 50 *μ*M	Increased p53 expression, p21, and caspase-3 activation, Bax, and Fas/APO1 expression Decrease in Bcl-2	[52]
*FaDu, hepatoma Hep 3B MG-63*	60 *μ*M	Induction of ERK and caspase-8	[53]
*Hep G2*	40 *μ*M	Overexpression ASK1, activation of JNK and MAPK	[82]
*WiDr*	30 *μ*mol/L	Caspase-9 and caspase-6 activation Downregulation of phosphorylated ERK Increased phosphorylation of p38 and SAPK/JNK	[75]
*DLD-1 and WiDr*	0.37 mM	Increased release of Cyt c and AIF Caspase-3 activation, DNA fragmentation, nuclear shrinkage	[76]
*A375*	40 *μ*M	Decreased Bcl-2 expression, downregulation of iNOS	[56]
*AGS and NCI-N87*	0.15 mM	Upregulation of TNF-*α* and Fas ligand Caspase-8 activation	[77]
*A431* *SCC25*	25.9 *μ*M 19.3 *μ*M	Increase in fragmented nuclei, the release of AIF and Cyt c Caspase-3 activation	[57]
*SGC-7901*	10 *μ*M	Increase in the caspase-3 and caspase-9 protein levels	[78]
*MCF7*	5, 10, 15, and 20 *μ*M	Induction of mitochondrial and ER death pathways	[30]
*MCF7*	100 *μ*M	Increased Fas expression	[79]
*MCF7*	80 *μ*M	Downregulation of IGFBP-2, IGF-1R, and B-raf gene expressions	[80]
*SkBr3*	40 *μ*M	Increase in cleaved PARP Suppressive effect on the PI3K/Akt/mtor signal pathway	[81]
*SK-N-BE(2c)* *SJ-N-KP*	5-30 *μ*M	Increase in the mRNA expression of p53 Increase in the p21, Bax, Bcl-2, and CD95 mRNA levels in SJ-N-KP cells but not in SK-N-BE(2c) cells	[69]
*K-562*	60 *μ*M	Increase in the active caspase-9 and procaspase-8 expression (12 h) Cleavage of procaspase-8 protein to the active forms (24 h) Increase in the active form of caspase-3 (72 h)	[44]
*P3HR-1*	28 *μ*M	Cleavage of procaspase-3, procaspase-9, and procaspase-8 proteins to the active forms (12 h)	[44]

## Data Availability

The data used to support the findings of this study are all included and available within the article.
